# Allelic variation of vernalization and photoperiod response genes in a diverse set of North American high latitude winter wheat genotypes

**DOI:** 10.1371/journal.pone.0203068

**Published:** 2018-08-30

**Authors:** Alexander Whittal, Mina Kaviani, Robert Graf, Gavin Humphreys, Alireza Navabi

**Affiliations:** 1 Department of Plant Agriculture, University of Guelph, Guelph, Ontario, Canada; 2 Agriculture and Agri-Food Canada, Lethbridge Research Centre, Lethbridge, Alberta, Canada; 3 Agriculture and Agri-Food Canada, Ottawa Research and Development Centre, Ottawa, Ontario, Canada; Institute of Genetics and Developmental Biology Chinese Academy of Sciences, CHINA

## Abstract

The major physiological determinants of wheat (*Triticum aestivum* L.) phenology in a given area are a response to vernalization temperature and day length, which are at least in part, regulated by the allelic variation at the vernalization (*VRN*) and photoperiod (*PPD*) loci, respectively. Characterization of the existing genetic variation for plant phenology in winter wheat can assist breeding programs improve adaptation to local environments and to optimize wheat phenology for the changing climate. The objectives of this research were to characterize the allelic variation at the major *VRN* and *PPD* loci in a diverse panel of high latitude winter wheat genotypes (n = 203) and to associate the allelic variation with phenologic, agronomic and adaptation traits. The panel was genotyped using allele-specific markers at vernalization (*VRN-A1*, *VRN-B1*, *VRN-D1* and *VRN-B3*) and photoperiod (*PPD-A1*, *PPD-B1*, and *PPD-D1*) loci and phenotyped for agronomically-important traits. Though photoperiod sensitivity was more prevalent, most of the variation in the phenology of the winter wheat panel was explained by allelic variation at *PPD-D1*, *PPD-A1*, and the interaction between these loci. While a typical high latitude winter wheat genotype is one that carries winter alleles at all major *VRN* loci and photoperiod sensitive alleles at the major *PPD* loci, in lower latitudes where winters are milder, the presence of one or two photoperiod insensitive alleles seems to contribute to higher yield and wider adaptation.

## Introduction

Wheat (*Triticum aestivum* L.) and other related temperate cereal species are able to grow under a wide range of agro-climatic regions [1]. A key factor underlying this successful wide adaptation is the variability in timing of important biological events that provides stress avoidance capabilities during different seasons [2]. For example, wheat can optimally coordinate flowering time with changing season to avoid freezing temperatures, heat stress and drought stress that could potentially damage the floral organs [2,3,4]. Local breeding programs can take advantage of the genetic variability governing these adaptive mechanisms to select for cultivars that suit their existing growing environment; thereby, developing cultivars resilient to future climate changes [5].

Flowering time in wheat is determined with two basic and well-described environmental cues: low temperature and photoperiod, which categorize wheat genotypes into winter or spring (with or without vernalization requirement) and photoperiod insensitive and sensitive [6]. The major genetic factors influencing such phenological characteristics in wheat are vernalization response genes (*VRN*), controlling the requirement of a cold period to switch from the vegetative to reproductive phase [7], as well as photoperiod sensitivity genes (*PPD*), determining plant response to day length [8,9].

Three major genes, *VRN-1*, *VRN-2* and *VRN-3* control the vernalization requirement in wheat. *VRN-1* and *VRN-3* induce flowering when dominant, with *VRN-1* having the main influence on the transition of the apex from vegetative to reproductive phase, while recessive mutants of *VRN*-2 accelerate flowering [10,11,12,13,14,15]. The *VRN-1* gene series include three homeologous loci *VRN-A1*, *VRN-B1*, and *VRN-D1*, on the long arm of chromosomes 5A, 5B, and 5D, respectively [8,10,16]. Greater polymorphism within the promoter, exon1 and intron1 regions has been reported for VRN-A1 compared to *VRN-B1* and *VRN-D1* [11,12,17]. Notably, mutations at the *VRN-A1* promoter region (*Vrn-A1a*) and intron 1 (*Vrn-A1b* and *Vrn-A1c*) and large deletions in intron 1 of the *VRN-B1* and *VRN-D1* genes have been associated with spring growth habit, whereas the presence of intact homozygous recessive *vrn-A1* allele confers winter growth habit [11,12].

Once vernalization requirement of winter wheat is fulfilled, photoperiod response will control the flowering time [2]. Photoperiod response in wheat is mainly controlled by the *PHOTOPERIOD1 (PPD-1)* loci located on the short arms of chromosomes 2A, 2B, and 2D (8). *PPD-1* genes identified in wheat and barley are members of the pseudo-response regulator (PRR) family [18,19]. Each locus has alleles that determine whether the plant has a photoperiod sensitive (long day) or insensitive (day-neutral) phenotype. Genotypes containing a photoperiod insensitive allele (suffix “*a*”, e.g., *Ppd-A1a*) will flower regardless of the duration of daylight, however, genotypes that are photoperiod sensitive (suffix “*b*”) will be delayed during short days and switch to reproductive stage when day length is increasing. Photoperiod insensitivity is mainly controlled by the dominant *Ppd-D1a* allele, followed by the *Ppd-B1a* and *Ppd-A1a* alleles [6,9].

Combinations of alleles at the *VRN-1* and *PPD-1* loci have been reported to result in variation in agronomic traits and physiological development such as flowering time [20,21,22], tillering, spikelet number, and plant height [9,23,24].

The influence of allelic variation at the *VRN-1* and *PPD-1* loci on heading date has been studied in Western regions of Canada for spring wheat collections [25,26,27] and several spring and winter wheat collections in other countries [6,28,29]. Most of the variation in heading date in spring wheat collections can be explained by allelic variation at *VRN-1* loci [25,26,27]. In contrast, *Ppd-D1*, *Ppd-B1* and their interaction were responsible for the variation in heading date for winter wheat collections studied in U.S. Great Plains [29]. A study of 683 lines from Europe, Asia, Africa, America, and Australia, reported that the winter and spring alleles at the *VRN-D1* locus had no significant effect on heading [6]. The study also examined the effect of photoperiod sensitive and insensitive alleles at the *PPD-B1* and *PPD-D1* loci, which had significant effects on heading date, with the photoperiod sensitive lines exhibiting later heading.

Due to the importance of these loci for their influence on flowering time, studying the allelic variation at these loci in relation to plant phenology will help determine which allelic combinations are most beneficial in a particular growing region or in optimizing plant phenology for the changing climate. Determining the optimal allelic combinations will facilitate marker assisted selection within breeding programs to avoid advancing undesirable allele combinations. The objectives of this study were to (i) determine the genetic variation at the *VRN* and *PPD* loci in a diverse set of wheat genotypes and (ii) study the plant phenology as influenced by *VRN/PPD* genotypes in fall-grown crop in high latitude winter wheat growing regions in Ontario, Canada. These analyses will provide a better understanding of the role of vernalization and photoperiod genes in determining the flowering and maturity times of winter wheats in northern latitudes.

## Materials and methods

### Plant material

The plant material consisted of a diversity panel of 203 winter wheat genotypes in two sub-panels. The first sub-panel, designated VRN144, consisted of 144 entries, including lines that are currently grown as winter wheats adapted to Canada (70 entries), and other parts of the world (3 entries), as well as elite lines (66 entries) currently in advanced stages of testing in breeding programs or in regional performance trials in Ontario, Canada. The second panel, designated VRN64, consisted of commercial winter wheat cultivars from the Canadian Prairies (39 entries) and 25 cold-tolerant spring wheat genotypes developed at the Lethbridge Research and Development Centre (LeRDC), Agriculture and Agri-Food Canada (AAFC) in Alberta, Canada. The cold-tolerant spring genotypes were developed directly from winter by spring crosses or by intercrossing previously characterized cold-tolerant spring wheat lines. Selection for spring growth habit and superior survivability occurred over several generations. Five genotypes were common between the two sub panels, containing a total of 203 unique genotypes.

### Environments and experimental design

In 2014–15, the entries were evaluated at the University of Guelph Elora Research Station near Elora, Ontario, Canada (43°38′N 80°25′W). The VRN144 sub panel was set up in a 12×12 partially balanced square lattice design with two replicates. The VRN64 sub panel was planted in the same field in an 8×8 partially balanced square lattice design with two replications. Each experimental unit was a two-row plot planted at a density of 400 seeds m^-2^,1.5m long with 17.8 cm row spacing and a 0.5 m alley separating plots, with a 35 cm space between adjacent plots.

During the 2015–2016 season, the diversity panel was planted in two locations; the Elora Research Station and the Woodstock Research Station (43°15′N 80°78′W) near Woodstock, Ontario, Canada, respectively. The same statistical designs were used as the 2014–2015 growing season. Each experimental design was a six-row plot 4m long, with 17.8 cm row spacing and with a 2 m alleyway separating plots.

### Phenotypic evaluation

Winter survival data were recorded in April on a 0 to 10 scale, where 0 indicates no plants survived and 10 indicates 100% of the plants survived. Crop developmental stages (booting, heading and anthesis) were determined for each field plot using Zadoks’ scale [30]. Number of days to booting (stage 41) was recorded when 75% of the tillers in the plot had the flag leaf fully expanded, the flag leaf sheath started opening and the head became visible inside the sheath. Number of days to heading (stage 59) was recorded when 75% of the tillers in the plot had complete head emergence. Number of days to anthesis (stage 61) was recorded when 75% of the tillers in the plot had anthers extruded from the florets. Number of days to physiological maturity (stage 87) was recorded when peduncles of 75% of the tillers in the plot turned color. Grain filling period (GFP) was measured by subtracting the days to anthesis from days to maturity. Plots were harvested with a Wintersteiger combine (Wintersteiger, Ried im Innkreis, Austria). Grain yield, test weight, and moisture content of each plot was collected at harvest using HarvestMaster’s Grain Gage (Juniper Systems, Inc., Logan, UT). Regardless of the range of maturity that was present among the genotypes, all plots were harvested at the same time, as there was no instance, in which an early variety may shatter. Plot yield data was then adjusted to 14% moisture content for every experimental unit according to the moisture measured at the time of harvest.

### Genotyping

#### PCR amplification of allele-specific markers

DNA was extracted from freshly grown seedlings. Four plants of each genotype, grown for a week in 96 well trays with cotton balls, were watered daily for five days. Five days after germination leaf tissue from the four plants was cut into smaller pieces and ground using an Eppendorf blue micro-pestle (Eppendorf, Hamburg, Germany) in a 1.5 ml tube. DNA extraction followed the Cetyl Trimethyl Ammonium Bromide (CTAB) protocol [31]. The quality and quantity of DNA were assessed by determining A260nm and A280nm using a NanoDrop (ND-1000) spectrophotometer (NanoDrop Technologies, Wilmington, DE, USA).

Genotyping was conducted using 7 allele specific PCR markers (Table 1). The primer set VrnN_FP3/R3 was designed by Primer Express Software (Applied Biosystems). PCR assays were carried out in Fisherbrand 96-well semi-skirted PCR plate 96-well Plates (Thermofisher, Mississauga, ON, Canada) using a Mastercycler Pro (Eppendorf, Hamburg, Germany) with 25 μl reactions consisting of 3–4 μl of 50 ngμl^-1^ template DNA, and 1X Taq PCR Master Mix (Qiagen, Maryland, USA). PCR cycles were conducted according to the conditions stated in the published reports (Table 1). The PCR products were size fractionated and visualized using the QIAxcel Advanced System (QIAGEN GmBH, Hilden, Germany).

**Table 1:** Allele-specific markers for alleles at the major vernalization and photoperiod response genes, primer sequence, PCR product size, annealing temperature and citation for the markers used in this study for the use of genotyping.

Locus	Allele (s)	Primer name	Sequence [5’-3’]	Product Size (bp)	Annealing Temp (°C)	Reference
*PPD-A1*		TaPpd-A1prodelF	CGTACTCCCTCCGTTTCTTT		57	32
	*Ppd-A1a*	TaPpd-A1prodelR3	AATTTACGGGGACCAAATACC	338 (*Ppd-A1a*)		
	*Ppd-A1b*	TaPpd-A1prodelR2	GTTGGGGTCGTTTGGTGGTG	299 (*Ppd-A1b*)		
*PPD-B1*	*Ppd-B1a*	TaPpd-B1proinF1	CAGCTCCTCCGTTTGCTTCC	650 (*Ppd-B1a*)	60	32
	*Ppd-B1b*	TaPpd-B1proinR1	CAGAGGAGTAGTCCGCGTGT	312 (*Ppd-B1b*)		
*PPD-D1*		Ppd-D1_F1	ACGCCTCCCACTACACTG		54	19
	*Ppd-D1a*	Ppd-D1_R2	CACTGGTGGTAGCTGAGATT	288 (*Ppd-D1a*)		
	*Ppd-D1b*	Ppd-D1_R1	GTTGGTTCAAACAGAGAGC	414 (*Ppd-D1b*)		
*VRN-A1*	*Vrn-A1a*	VrnN_FP3	GTGTGTGTTTGTGGCGAGAG	926 (*Vrn-A1a*)	55	in this study ^1^
	*Vrn-A1b*	VrnN_RP3	CGAAGGCGTATTGGGGAACA	633 (*Vrn-A1b*)		
	*vrn-A1*			662 (*vrn-A1*)		
*VRN-B1*	*Vrn-B1a*	Intr/B/F	CAAGTGGAACGGTTAGGACA	709 (*Vrn-B1a*)	58	12
	*Vrn-B1b*	Intr1/B/R3	CTCATGCCAAAAATTGAAGATGA	673 (*Vrn-B1b*)		
	*vrn-B1*	Intr/B/F	CAAGTGGAACGGTTAGGACA	1149	56.4	12
		Intr1/B/R4	CAAATGAAAAGGAATGAGAGCA			
*VRN-D1*	*Vrn-D1*	Intr1/D/F	GTTGTCTGCCTCATCAAATCC		61	12
		Intr1/D/R3	GGTCACTGGTGGTCTGTGC	1671		
	*vrn-D1*	Intr1/D/R	AAATGAAAAGGAACGAGAGCG	997		
*VRN-B3*	*Vrn-B3*	VRN4-B-INS-F	CATAATGCCAAGCCGGTGAGTAC	1200	57	10
		VRN4-B-INS-R	ATGTCTGCCAATTAGCTAGC			
	*vrn-B3*	VRN4-BNOINS-F	ATGCTTTCGCTTGCCATCC	1140	57	10
		VRN4-BNOINS-R	CTATCCCTACCGGCCATTAG			

^1^ Figure A in S1 File

### Data analysis

#### Cluster analysis

Genotypes that had heterogeneous or undetermined alleles at any locus were removed from the cluster analysis. Genotypic data for the 203 genotypes was subjected to cluster analysis. Genotypic data was imported into the software Graphical Genotype (GGT 2.0; [32]), in which a matrix of the pair-wise genetic distances were computed. This matrix was then saved as a MEGA file. The dissimilarity matrix was then exported to the MEGA 7.0 software [33] where an unweighted pair group method with arithmetic mean dendrogram was calculated and a dendrogram was generated using”Construct/Test UPGMA Tree” command under the phylogeny tab.

#### Growing degree days

Cumulative growing degree-days (CGDD) was calculated as the number of daily growing degree days received to reach various phenological stages (booting, heading, anthesis, and physiological maturity) using the formula explained by McMaster and Smika, 1988 [34]. The equation for calculating GDD is:

$$GGD=\sum_{i=1}^{p}[(Daily\;Max\;Temp+Daily\;Min\;Temp)÷2]-4$$

where *i* is the number of the day ranging from the first frost-free day in each season (April 10^th^ in Elora 2015 and April 15^th^ in 2016 trials) to the *p*^th^ day, in which the respective phenological stage was recorded.

#### Analysis of variance

Analyses of variance of the phenotypic data were conducted using the PROC MIXED procedure in SAS 9.4 (SAS Institute, Inc., Cary, NC). The mixed model analysis was conducted on the raw data to determine significance of fixed (genotype) and random (block, incomplete bloc within block, environment, and their interactions) factors. Least squared means (Lsmeans) for the genotypes were computed using LSMEANS statement. Tests of normality of residuals were performed using Shapiro Wilk [35], Kolmogorov-Smirnov [36,37], Cramer-von Mises [37], and Anderson-Darling [38,39] in PROC UNIVARITA procedure of SAS. The scatter plot of studentized residuals against predicted values were generated in PROC GPLOT to examine the random and independent distribution of residuals.

Statistical significance of the genotypic groups at each of the *VRN* and *PPD* loci were examined using a linear mixed model in the PROC MIXED procedure of SAS, in which the phenotypic value was examined as a linear function of the genotype at each locus. If genotypes were heterogeneous or unidentified for a certain locus then they were removed from the analysis of the locus. The loci *PPD-B1*, *VRN-B1*, *VRN-D1* and *VRN-B3* were not tested against phenological traits due to a lack of variation at these loci. Pearson’s coefficients of correlations of phenological traits were computed using the PROC CORR Procedure in SAS.

Principle component analysis was performed to examine the relationships between the observed traits using the PROC PRINCOMP and PRINQUAL in SAS. Biplots were created using PC1 and PC2 values as the x and y-axis, respectively.

Box plots were created in Sigma-plot software (Systat Software Inc., Richmond, CA), using the box plot function. The whiskers of the box plot extend out to the 10^th^ and 90^th^ percentiles. Data points that extend past the whiskers are considered outliers.

## Results

### Genotyping

#### Allelic frequency at the *VRN-1* and *PPD-1* loci

Analysis of the diversity panel (n = 203) with 7 gene-specific markers (Table 1) revealed that *PPD-D1* locus had the most allelic variation among the three photoperiod sensitivity loci, with 127 genotypes carrying the photoperiod-sensitive *Ppd-D1b* allele and 68 genotypes carrying the photoperiod insensitive *Ppd-D1a* allele (Table 2). The locus with the second largest variation was *PPD-A1*, in which 170 genotypes carried the photoperiod sensitive *Ppd-A1b* allele and 31 genotypes carried the photoperiod-insensitive *Ppd-A1a* allele (Table 2). The locus *PPD-B1* did not have any allelic variation, such that all 203 genotypes carried the photoperiod sensitive *Ppd-B1b* allele (Table 2).

**Table 2:** Frequency of genotypes with different photoperiod and vernalization alleles at the major loci for 203 genotypes in the diversity panel.

Number of Genotypes (%)			
Locus	Insensitive^a^/Spring^b^	Sensitive^a^/Winter^b^	Heterogeneous
*PPD-A1*	31 (15.0%)	170 (84.0%)	2 (1.0%)
*PPD-B1*	0	203 (100.0%)	0
*PPD-D1*	68 (33.5%)	127 (62.5%)	8 (4.0%)
*VRN-A1*	18 (9.0%)	182 (89.0%)	2 (1.0%)
*VRN-B1*	1 (0.5%)	201 (99.0%)	1 (0.5%)
*VRN-D1*	1 (0.5%)	202 (99.5%)	0
*VRN-B3*	0	203 (100.0%)	0

a: insensitive /sensitive for PPD genes

b: Spring/winter for VRN genes

*VRN-A1* showed more variability compared to other major vernalization loci tested, with 182 genotypes carrying the recessive *vrn-A1* winter allele, and 18 genotypes carrying the *Vrn-A1a* spring allele (Table 2). With only an exception of one genotype carrying spring *Vrn-B1* allele, the rest of the germplasm (201 entries) carried the recessive *vrn-B1* allele (Table 2). Low variation was found for *VRN-D1* locus. Most of the genotypes (202 entries) carried the recessive *vrn-D1* winter allele; only one genotype carried the spring *Vrn-D1* allele (Table 2). The germplasm did not have any variation at the *VRN-B3* locus, with all 203 genotypes carrying the recessive *vrn-B3* allele (Table 2).

#### Cluster analysis

**Fig 1:**
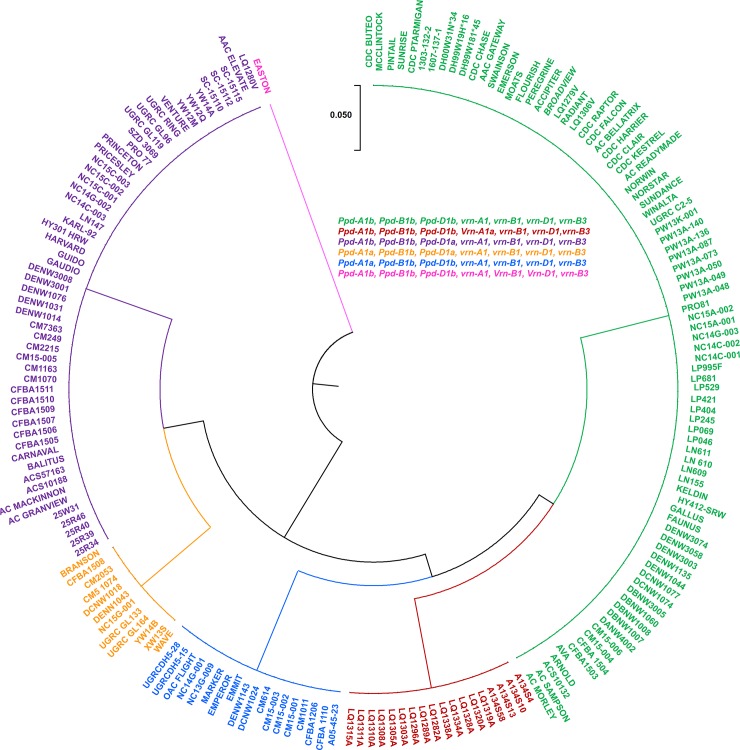
Dendrogram of genotype loci combinations. In clock wise order*; Ppd-A1b/ Ppd-B1b/Ppd-D1b/vrn-A1/vrn-B1/ vrn-D1/vrn-B3* (green), *Ppd-A1b/Ppd-B1b/Ppd-D1b/Vrn-A1a/vrn-B1/vrn-D1/vrn-B3* (red), *Ppd-A1a/Ppd-B1b/Ppd-D1b/vrn-A1/vrn-B1/vrn-D1/vrn-B3 (blue)*, *Ppd-A1a/Ppd-B1b/Ppd-D1a/vrn-A1/vrn-B1/vrn-D1/vrn-B3* (yellow), *Ppd-A1b/Ppd-B1b/Ppd-D1a/vrn-A1/vrn-B1/vrn-D1/vrn-B3* (Purple), *Ppd-A1b/Ppd-B1b/Ppd-D1b/vrn-A1/Vrn-B1/Vrn-D1/ vrn-B3* (Pink).

Cluster analysis of the 203 genotypes identified six distinct clusters, based on their genotypes at the major *VRN* and *PPD* loci, using allele-specific marker data (Fig 1). The largest cluster (shown in green, Fig 1; n = 85) consisted of genotypes with photoperiod-sensitive alleles at all three major *PPD* loci, *Ppd-A1b/Ppd-B1b/Ppd-D1b*, and the recessive winter alleles at all three *VRN-1* loci, *vrn-A1/vrn-B1/vrn-D1* as well as the winter allele *vrn-B3*. The second largest cluster (shown in purple, Fig 1; n = 55) had the photoperiod-insensitive allele *Ppd-D1a*, the photoperiod-sensitive *Ppd-A1b* and *Ppd-B1b* alleles, and vernalization-sensitive *vrn-A1*, *vrn-B1*, *vrn-D1*, and *vrn-B3* alleles at the *VRN-1* loci (Fig 1).

The next cluster (shown in blue, Fig 1; n = 18) had the photoperiod-insensitive allele *Ppd-A1a*, and the sensitive alleles *Ppd-B1b* and *Ppd-D1b*, as well as the recessive winter alleles; *vrn-A1*/*vrn-B1*/*vrn-D1/ vrn-B3*. Another cluster (shown in yellow, Fig 1; n = 12) had the photoperiod-insensitive alleles *Ppd-A1a* and *Ppd-D1a*, but the sensitive *Ppd-B1b* allele and the winter-sensitive alleles *vrn-A1*, *vrn-B1*, *vrn-D1*, and *vrn-B3* (Fig 1). Most of the Canadian winter wheat varieties are grouped in these four clusters; all carried recessive winter alleles at the *VRN*-*A1*, *VRN-B1*, *VRN-D1*, and *VRN*-*B3* loci in addition to photoperiod-sensitive alleles at either one, two or all three major *PPD* loci (Fig 1).

The remaining genotypes carried at least one spring allele at one of the three major *VRN* loci. The largest cluster of this kind that contained spring alleles (shown in red, Fig 1; n = 18), had the photoperiod-sensitive alleles *Ppd-A1b*, *Ppd-B1b*, and *Ppd-D1b*, and winter-sensitive alleles *vrn-B1*, *vrn-D1*, and *vrn-B3* but the spring allele *Vrn-A1a*. These genotypes are exclusively the winter-hardy spring lines from Alberta, Canada (Fig 1), which were selected for maintenance of fall vegetative growth and spring growth habit, along with winter hardiness. The sixth and smallest cluster (shown in pink, Fig 1; n = 1) is a spring wheat check in the test, which contained the photoperiod sensitive alleles *Ppd-D1b* and *Ppd-A1b*, as well as the winter alleles *vrn-A*1 and vrn-B3, but had the spring alleles; *Vrn-B1* and *Vrn-D1* loci (Fig 1).

#### Effect of the allelic variation at the *VRN-1* and *PPD-1* loci on phenotypic traits

**Fig 2:**
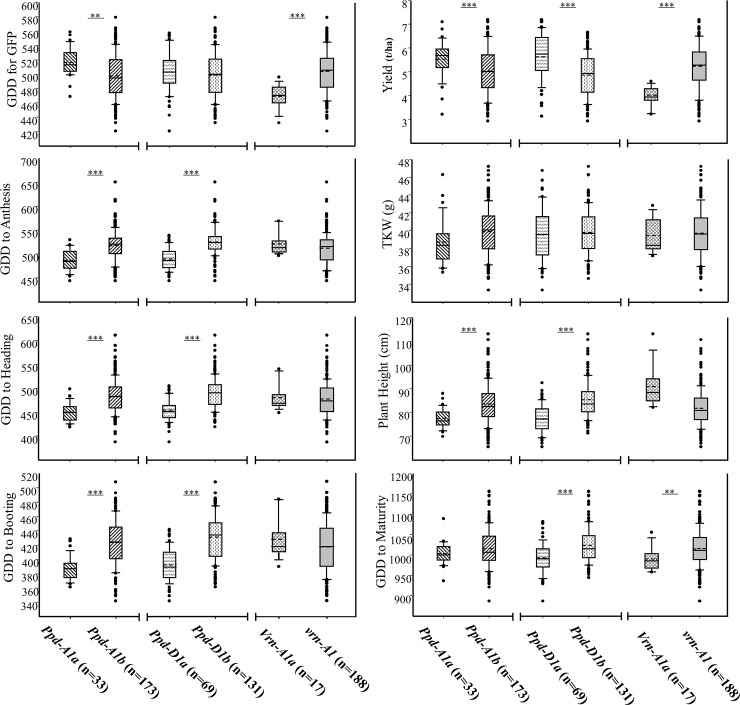
Box plots representing the frequency distribution of genotypes in three *Ppd-A1*, *Ppd-D1* and *Vrn-A1* genotypic groups for phenotypic traits observed in the field trials during 2015 and 2016 in Ontario, Canada. *, **, and *** represent significant differences at 0.05, 0.01, and 0.001, respectively. n = Number of genotypes in each group. In each box-plot, solid dots represent outliers. Each box represents the interquartile range, which contains 50% of the values. The whiskers are lines that extend from the box to the highest and lowest values, excluding outliers. The dashed and solid lines across the box indicate the median and mean, respectively. GDD: Growing Degree Days. TKW: Thousand-Kernel Weight (g). GFP: Grain Filling Period.

Significant differences (P = <0.001) were observed among genotypes for yield, plant height, GFP, Thousand-Kernel Weight (TKW) and for the number of growing degree days required to reach booting, heading, anthesis, and maturity (Table 3). The interaction effect of genotype-by-environment (G×E) was significant (P = <0.001) for all traits, suggesting that genotypes responded differently to environments (Table 3). The phenotypic differences of the genotypes with different alleles at the *VRN* and *PPD* loci were examined in separate mixed model analyses (Table 3). *Ppd-D1b* and *Ppd-A1b* were associated with 38.78 and 36.52 GDDs later booting in combined analysis across environments as compared to the photoperiod insensitive lines carrying *Ppd-D1a* and *Ppd-A1a*, respectively (Fig 2). This pattern repeated itself for GDDs to heading and anthesis for entries with insensitive alleles at the *PPD-D1* and *PPD-A1* loci (Table 3, Fig 2). The average effect of *Ppd-D1b* was 36.29 GDDs later to heading and 34.21 GDDs later for entries with *Ppd-A1b* compared with entries with insensitive alleles (Table 3, Fig 2). In contrast, genotypes with *Ppd-D1b* reached anthesis 35.68 days later, while entries with *Ppd-A1b* reached anthesis 31.19 days later than genotypes with the insensitive alleles (Table 3, Fig 2). Genotypes with different alleles at the *VRN-A1* locus, however, were not different for the number of GDD to booting, heading and anthesis (Table 2, Fig 2).

**Table 3:** Mixed model analysis of variance of 208 genotypes in a diversity panel for phenotypic traits observed in 2015 and 2016 field trials planted in Elora and Woodstock Ontario locations. (Fixed effect DF = 207, Loci effect DF = 1).

	GDD ^1^ to Booting		GDD to heading	GDD to Anthesis	GFP ^2^	GDD to maturity	Yield	TKW ^3^	Height
	*F*-Value		*F*-Value	*F*-Value	*F*-Value	*F*-Value	*F*-Value	*F*-Value	*F*-Value
*Fixed effect*									
Entry	12.33 ***		11.86***	13.11***	2.17***	3.66***	3.60***	1.85***	11.92***
	Estimate		Estimate	Estimate	Estimate	Estimate	Estimate	Estimate	Estimate
*Random effects*									
Environment	2651.25 ns ^4^		2991.42 ns	3808.15 ns	4867.41 ns	17151.00 ns	0.82 ns	116.44 ns	34.56 ns
Block (env)	5.15 ns		1.55 ns	3.97 ns	0.00	0.00	0.00	0.04 ns	1.42 ns
iBlock (env*Block)	18.69 ***		20.41 ***	18.25 **	379.58 ***	374.58 ***	0.27 ***	0.47 **	4.57 ***
Entry*Environment	197.32 ***		239.98***	155.60***	880.66***	1313.47***	0.30***	7.94***	8.59***
Residual	156.83 ***		149.57***	161.15***	681.43***	614.33***	0.38***	6.11***	13.62***
Loci	*F*-Value		*F*-Value	*F*-Value	*F*-Value	*F*-Value	*F*-Value	*F*-Value	*F*-Value
*Ppd-D1*	40.74***		30.65***	37.21***	0.68 ns	11.91***	16.73***	0.37 ns	28.15***
*Ppd-A1*	19.75***		15.47***	14.81***	5.61**	1.03 ns	4.20*	2.89 ns	8.03***
*Vrn-A1*	1.30 ns		0.96 ns	0.71 ns	14.99***	4.89**	15.09***	0.10 ns	12.09***
Ppd-D1*Ppd-A1	5.40**		2.87*	2.82*	0.15 ns	1.12 ns	0.70 ns	0.33 ns	0.59 ns

1-GDD: Growing Degree Days.

2-GFP: Grain Filling Period.

3-TKW: Thousand Kernel Weight.

4-ns: non-significance at the 0.05 probability level.

*, **, *** significance at the 0.05, 0.01, and 0.001 probability levels, respectively.

The effect of allelic variation at the *PPD-D1* locus was not significant for GDD required for GFP (Table 3, Fig 2). Genotypes with different alleles at the *PPD-A1* locus, on the other hand were significantly different for GDD required for GFP (Table 2). The average GFP of the genotypes with *Ppd-A1a* or *Ppd-A1b* were 516.47 and 497.44 GDD, respectively (Fig 2). Similarly, genotypes with different alleles at the *VRN-A1* locus were significantly (P = <0.0001) different for GDD required for GFP (Tables 2 and 3). The average GFP for genotypes with *Vrn-A1a* or *vrn-A1* were 469.15 and 504.09 GDD, respectively (Fig 2).

Genotypes with different alleles at the *PPD-D1* (P = <0.0001), *PPD-A1* (P = 0.01), and *VRN-A1* (P = <0.0001) loci were significantly different for yield (Table 3). The average yield of genotypes with *Ppd-D1a* or *Ppd-D1b* were 5.63 and 4.87 t ha^-1^, respectively (Fig 2), while genotypes with *Ppd-A1a* or *Ppd-A1b* on average yielded 5.53 and 5.03 t ha^-1^, respectively (Fig 2). The average yield of genotypes with *Vrn-A1a* or *vrn-A1* were 3.98 and 5.23 t ha^-1^, respectively (Fig 2).

Genotypes with different alleles at *PPD-D1* (P = <0.0001), *PPD-A1* (P = 0.0004), and *VRN-A1* (P = <0.0001) were significantly different for plant height (Table 3). The average plant height of genotypes with *Ppd-D1a* or *Ppd-D1b* allele were 77.35 and 85.40 cm, respectively (Fig 2), while the average plant height of genotypes with *Ppd-A1a* or *Ppd-A1b* alleles were 77.57 and 83.54cm, respectively (Fig 2). The average plant height of genotypes with *Vrn-A1a* or *vrn-A1* alleles were 90.86 and 81.73cm, respectively (Fig 2).

#### Correlations and principle component analysis

The GDD to anthesis was very closely associated with GDD to booting (r = 0.94) and GDD to heading (r = 0.96) in the combined year biplot. Anthesis and maturity were associated (r = 0.73), but GDD to anthesis and GFP had no association (r = 0.00) (Table 4). Maturity was associated with GFP (r = 0.68), but had no association with yield (r = 0.01). GFP and yield were closely associated (r = 0.35) (Table 4). Yield had a positive correlation with TKW (r = 0.3), but it had a negative association with plant height (r = -0.29) and GDD to anthesis (r = -0.32) (Table 4).

**Table 4:** Pearson’s coefficient of correlations (above) and their significance probability (below) for pair-wise phenotypic traits observed in 2015 and 2016 field trials in Elora and Woodstock locations.

	GDD ^1^ to Anthesis	GDD to Booting	GFP ^2^	GDD to Heading	Height	GDD to Maturity	TKW ^3^ (g)	Winter Survival
**GDD to Booting**	0.94							
	***							
**GFP**	0.00	0.00						
	ns ^4^	ns						
**GDD to Heading**	0.96	0.95	0.12					
	***	***	ns					
**Height**	0.41	0.50	-0.30	0.36				
	***	***	***	***				
**GDD to Maturity**	0.73	0.68	0.68	0.78	0.09			
	***	***	***	***	***			
**TKW (g)**	0.06	0.08	0.10	0.07	0.28	0.11		
	ns	ns	ns	ns	***	ns		
**Winter Survival**	-0.24	-0.08	-0.40	-0.26	0.28	-0.45	0.03	
	***	ns	***	***	***	***	ns	
**Yield (t ha**^**-1**^**)**	-0.32	-0.32	0.35	-0.27	-0.29	0.01	0.30	-0.04
	***	***	***	***	***	ns	***	ns

1-GDD: Growing Degree Days.

2-GFP: Grain Filling Period.

3-TKW: Thousand Kernel Weight.

4-ns: non-significance at the 0.05 probability level.

*** Significance at the 0.001 probability levels.

**Fig 3:**
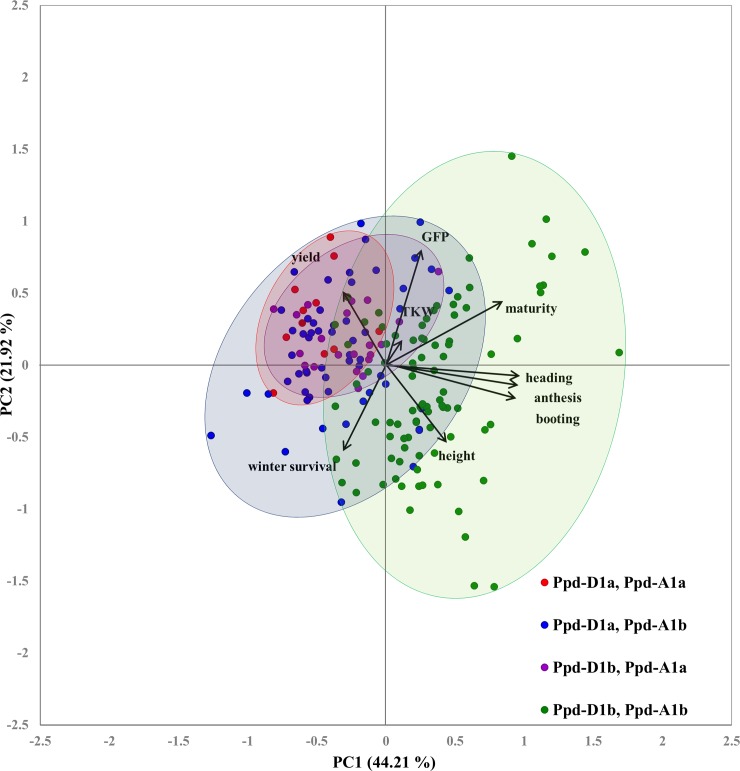
Biplot of the principle component analysis of the genotype by trait matrixes for 2015 and 2016 for 203 genotypes tested in fall-seeded trials in Ontario, Canada.

Principle component analysis (PCA) with GFP, TKW, height, yield, winter survival and phenological traits of 203 genotypes was performed to assess whether these variables could be used to differentiate varieties with different alleles at *PPD-D1* and *PPD-A1* (Fig 3). The first two principle components accounted for 66.13% of the variation. Component 1 was positively correlated with GFP, TKW, height and phenology traits and negatively correlated with yield and winter survival. In contrast, component 2 was only correlated with yield, GFP, TKW and maturity (Fig 3). The biplot of the first two principle components separated the effect of PPD sensitivity along the PC1 axis, with all photoperiod sensitive genotypes grouping togeather (with some overlap), clustering towards booting, heading, anthesis, maturity, GFP, plant height and TKW (Fig 3). In contrast genotypes with at least one photoperiod-insensitive allele clustered mainly in the left side of biplot tending towards higher yield, and away from phenological traits (Fig 3). Within genotypes with photoperiod insensitive alleles, fully insensitive ones showed more tendency towards yield (Fig 3).

## Discussion

Using diagnostic molecular markers for the most common alleles of the *VRN* and *PPD* loci affecting the vernalization requirement and photoperiod response, we characterized a germplasm collection of Canadian winter wheat genotypes grown or developed for the higher latitude regions of North America. The first objective of this study was to evaluate the allelic variation at the important *VRN* and *PPD* loci. Marker analysis revealed a high prevalence of photoperiod sensitivity alleles at the *PPD-D1* (62.5%), *PPD-A1* (84%) and *PPD-B1* (100%) loci. High frequency of *Ppd-D1* sensitive alleles in higher latitudes was previously reported by Guo et al., [40] in lines collected from Canada and U.S. in a worldwide wheat collection (n = 492). The photoperiod insensitive allele *Ppd-D1b* was present at levels similar levels to that detected by Kiss et al. [6] in winter wheat lines from America and Africa compared to those from other continents.

In the germplasm studied in this investigation, we observed a lack of variation at the *VRN* loci, which was comparable to low frequency (2%) of *VRN*-*A1* in a European winter wheat collection of 521 winter wheat genotypes [6]. The *Vrn-B1* and *Vrn-D1* spring alleles in their study were present at slightly higher frequencies compared to the Canadian winter wheat genotypes. Lack of variation at the major *VRN* loci was expected, considering that the diversity panel was almost exclusively winter wheat genotypes adapted to Canadian conditions.

The low variation at the *VRN-1* locus was also observed in a winter wheat collection (n = 299) from U.S. Great Plains [29]. However, they found higher frequency of photoperiod-insensitive alleles at *PPD-B1* (53%), rare occurrences of *Ppd-A1a* (2%), and comparable *Ppd-D1a* (35%). They indicated that varieties from the northern Great Plains had greater incidences of the photoperiod sensitive alleles than germplasm from central and southern breeding programs [29].

Many genetic studies demonstrated that winter and spring wheat cultivars grown in northern latitude countries usually carry photoperiod sensitive alleles at higher frequencies. In contrast, genotypes grown in southern latitudes normally carry photoperiod-insensitive alleles [9,28,29,41,42,43]. This is likely attributable to less variation in day length in southern latitude regions where photoperiod insensitivity could have clear adaptive significance [29,44].

The typical Canadian winter wheat material carried winter alleles at all major *VRN* loci and photoperiod-sensitive alleles at either three or two of the major *PPD* loci. Most genotypes had the *Ppd/Vrn* allelic combination of *Ppd-A1b*, *Ppd-B1b*, *Ppd-D1b*, *vrn-A1*, *vrn-B1*, *vrn-D1*, and *vrn-B3*, which accounted for 43% of genotype combinations. The second largest group was similar except it carried *Ppd-D1a* instead of *Ppd-D1b*, and accounted for 28% of genotypes. Genotypes with all three *VRN-1* winter alleles and either one or more of the *Ppd* insensitive alleles account for 42% of the total genotypes. This indicates that selection in the Canadian winter wheat breeding programs has favored selection for photoperiod-sensitivity allele(s) at the major *PPD* loci. Grogan et al. [29] also reported an increase in selection of photoperiod-insensitive alleles throughout the U.S. Great Plains after the year 2000. They found higher frequency of photoperiod sensitive alleles *Ppd-A1b*, *Ppd-B1b*, and *Ppd-D1b* northern plains compared to central and southern plains [29]. On the contrary, Kamran et al. [26] reported that in Western Canadian spring wheat, *Ppd-D1b* is being replaced with the photoperiod-insensitive *Ppd-D1a* allele in recent germplasm.

Our second objective was to study the implications of *VRN* and *PPD* variation on plant phenology in a diverse set of fall-sown winter and spring wheat lines that represent the genetic variation in winter wheat in higher latitudes (>40°N) of North America.

We found that day length sensitive photoperiod genes play a major role in determining flowering time and adaptability of Canadian winter wheat. Grogan et al. [29] found heading date in winter wheat from the U.S. Great Plains is strongly affected by photoperiod loci. Similar results were observed in present study, where genotypes with varying *Ppd-A1/D1* allele(s) had different time to booting, heading, anthesis, GFP, and height. As expected, photoperiod insensitivity resulted in earlier flowering compared to photoperiod sensitivity. On average, day length insensitive genotypes required 41.8 growing degree-days less than the genotypes with photoperiod-sensitive alleles at all three *PPD* loci to reach anthesis. The results of earlier flowering of photoperiod-insensitive genotypes is consistent with results from previous studies reporting 3.7 and 4.3 days earlier booting and heading, respectively [6], 1.6 to 8 days earlier flowering [29,45], conditioned by the presence of *Ppd-D1a* allele, when compared to photoperiod-sensitive genotypes.

Despite earlier anthesis, genotypes with the photoperiod-insensitive allele *Ppd-D1a* in general yielded 13.5% higher than the photoperiod-sensitive genotypes, when compared to the genotypes with *Ppd-D1b* allele. The difference in yield is consistent with other reports; for example, in southern Europe, increased yields of up to 35% were reported for the photoperiod-insensitive genotypes carrying the *Ppd-D1a* allele [9,45]. Similarly, 7.7% higher yield in Germany and 30% in the former Yugoslavia [9] were reported for genotypes with the *Ppd-D1a* allele. The higher yield in *Ppd-D1a* genotypes can be explained by escape from hot and dry summer days due to earlier flowering [9]. In this study, higher yield for *Ppd-D1a* genotypes may be linked to avoidance of biotic stresses associated with powdery mildew (*Erysiphe graminis*) and Fusarium head blight (caused mainly by *Fusarium graminearum*) which were present in 2015, and stripe rust (caused by *Puccinia striiformis*) in 2016 [42,45].

The difference in the genotypic groups at the *VRN-A1* locus was significant for GFP, height and time to maturity. The extension of GFP was due to a delay in maturity among the *vrn-A1* genotypes, while the two groups reached anthesis at the same time. The accelerated time to maturity of *Vrn-A1a* genotypes is consistent with a study of Canadian hard red spring wheat genotypes [26]. *Vrn-A1a* also induced earlier flowering, compared to *vrn-A1* [25, 26]; however, in this study days to anthesis was not significantly different between the two allelic groups [26]. The lower yield of Vrn-A1a genotypes (cold tolerant spring wheats) may be due to the panel having a high percentage of high-yielding winter wheat genotypes. However, based on the influence of time to maturity, *vrn-A1* genotypes with a delayed maturity may have demonstrated yield high due to a prolonged GFP. In addition, the cold-tolerant spring wheat genotypes were primarily selected on the basis of winter survival, with little regard for grain yield.

There have been several theories as to why the spring alleles at the *VRN* locus and the photoperiod insensitive alleles at the *PPD* loci induce earlier flowering. Davidson et al. [46] proposed that the accelerated flowering was due to developmental acceleration from emergence to floral initiation. Photoperiod insensitivity is associated with suppression of *PPD1* and up regulation of VRN3, which in turn promotes flowering by inducing meristem identity genes [47,48]. The earliness may also be induced by shortening the required heat unit accumulation between emergence and stem elongation [49]. Based on the results of the present study, a combination of the described theories can explain the differences of the time needed to reach booting, heading, and anthesis between photoperiod insensitive and sensitive genotypes.

## Conclusions

We studied a panel of 208 winter wheat genotypes representative of modern and historic Canadian winter wheat to evaluate allelic diversity and effects of vernalization and photoperiod loci on heading time as well as other physiological stages. We found that most of the variation in the phenology of winter wheat crop in higher latitudes can be explained by allelic variation at the *PPD-D1*, *PPD-A1*, and the interaction between these loci. Selecting for photoperiod insensitivity in the presence of winter alleles at the vernalization loci for fall-seeded wheat-growing regions in the high latitudes of the northern hemisphere may provide wider environmental adaptation. This is a result of early flowering due to photoperiod insensitivity, which results in higher yield due to avoiding late season biotic and /or abiotic stress factors. Increased frequency of photoperiod insensitive alleles in the winter wheat in the higher latitudes may become more prevalent with more frequent occurrence of milder winters due to climate change. Our study also indicates that breeding winter-hardy spring wheat genotypes seems achievable for milder winter areas by selecting for one spring allele at one of the *VRN-1* loci (*VRN-B1*, or *VRN-D1*) in combination with photoperiod sensitive alleles at all major *PPD* loci, which provides some protection of the floral meristem during the fall, along with an acceptable level of cold hardiness.

## Supporting information

S1 File New VRN-A1 primers. Sequence used for designing new *VRN-A1* primers and QIAxcel gel image showing PCR products for new *VRN-A1* primers.
(XLSX)

S2 File Field and genotyping data. Phenology, yield and genotyping raw data.
(PDF)
