# Correction: Allelic variation of vernalization and photoperiod response genes in a diverse set of North American high latitude winter wheat genotypes

**DOI:** 10.1371/journal.pone.0209543

**Published:** 2018-12-17

**Authors:** The PLOS ONE Staff

S1 and S2 Files are incorrectly swapped. The publisher apologizes for this error. Please see the corrected files here.

## Supporting information

S1 File New *VRN-A1* primers. Sequence used for designing new *VRN-A1* primers and QIAxcel gel image showing PCR products for new *VRN-A1* primers.
(PDF)

S2 File Field and genotyping data. Phenology, yield and genotyping raw data.
(XLSX)
