# Supplementary material for: Allelic variation of vernalization and photoperiod response genes in a diverse set of North American high latitude winter wheat genotypes
Source: PLoS One. 2018 Aug 30;13(8):e0203068. doi: 10.1371/journal.pone.0203068 (PMC6117032; doi:10.1371/journal.pone.0203068)
Supplement: S2 File — Phenology, yield and genotyping raw data. (PDF) [file pone.0203068.s002.pdf]

Supporting Information 1

Sequence used for New VRN-A1 primers

>AY747601.1

    >>>>Forward

….GTGTGTGTTTGTGGCGAGAGAAAATGATTTGGGGAAAGCAAAATCCGGAGATTCGCACGTACGATC
GTTCGACACGTCGACGCCCGGCGGGCCCGGGGTGGGGCATCGTGTGGCTGCAGGACCGCGGGGCCCC
GCAAAGCGGGCCGGGCCAATGGGTGCTCGACAGCGGCTATGCTCCAGACCAGCCCGGTATTGCATACC
GCGCTCGGGGCCAGATCCCTTTAAAAACCGGAAAAAAATTATATGAGACCAGGTCTCATATAAATCAG
GTGAGACCCGCCCTGATGAATGACATGTGGCATTCACAAATCACAAAGCATCTAATCTCTCCCCCCCT
GATTTCAGGTGGGGGGTGGGGTGGATGCTTTGTGATTTGTGAATGACACGTGTCATCCATCAGGATGG
GTCTCACCTGCTAATCCGTGAGACCTGGTCTCATAGAATTTTTTTCCTTAAAAACCCCTCCCCCCCTGCC
GGAATCCTCGTTTTGGCCTGGCCATCCTCCCTCTCCTCCCCTCTCTTCCACCTCACGTCCTCACCCAACC
ACCTGATAGCCATGGCTCCGCCGCCTCGCCTCCGCCTGCGCCAGTCGGAGTAGCCGTCGCGGTCTGCC
GGTGTTGGAGGGTAGGGGCGTAGGGTTGGCCCGGTTCTCGAGCGGAGATGGGGCGGGGGAAGGTGCA
GCTGAAGCGGATCGAGAACAAGATCAACCGGCAGGTGACCTTCTCCAAGCGCCGCTCGGGGCTTCTCA
AGAAGGCGCACGAGATCTCCGTGCTCTGCGACGCCGAGGTCGGCCTCATCATCTTCTCCACCAAGGGA
AAGCTCTACGAGTTCTCCACCGAGTCATGGTAAATTAAGCACGCGCTGTCTTTAAATTTGTTCCCCAAT
ACGCCTTCG ….

Reverse <<<<<<

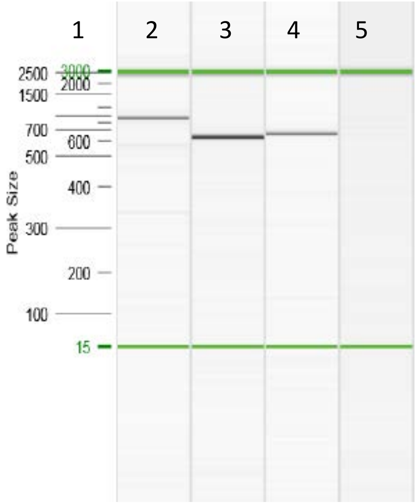

Figure A: QIAxcel gel image showing PCR products for new Vrn-A1 primers. Lane 1:100-2500 bp size marker aligned with 15-3000 bp alignment marker (green lines), 2: Red Fife (*Vrn-A1a*) (~920bp), 3: Ac Meena (*Vrn-A1b*) (~630 bp), 4: Ac Crystal (*vrn-A1*) (~662bp) and 5: No Template Control.
